# Efficient propagation of archetype JC polyomavirus in COS-7 cells: evaluation of rearrangements within the NCCR structural organization after transfection

**DOI:** 10.1007/s00705-017-3542-7

**Published:** 2017-09-07

**Authors:** Carla Prezioso, Daniela Scribano, Anna Bellizzi, Elena Anzivino, Donatella Maria Rodio, Maria Trancassini, Anna Teresa Palamara, Valeria Pietropaolo

**Affiliations:** 1 Department of Public Health and Infectious Diseases, “Sapienza” University, P.le Aldo Moro, 5, 00185 Rome, Italy; 2 Department of Experimental and Clinical Sciences, “G. D’Annunzio” University of Chieti, Chieti, Italy; 3 Department of Public Health and Infectious Diseases, Institute Pasteur, Cenci-Bolognetti Foundation, Sapienza University of Rome, Rome, Italy; 4 San Raffaele Pisana Scientific Institute for Research, Hospitalization and Health Care, Rome, Italy

## Abstract

John Cunningham virus (JCPyV) is an ubiquitous human pathogen that causes disease in immunocompromised patients. The JCPyV genome is composed of an early region and a late region, which are physically separated by the non-coding control region (NCCR). The DNA sequence of the NCCR distinguishes two forms of JCPyV, the designated archetype and the prototype, which resulted from a rearrangement of the archetype sequence. To date, the cell culture systems for propagating JCPyV archetype have been very limited in their availability and robustness. Prior to this study, it was demonstrated that JCPyV archetype DNA replicates in COS-7 simian kidney cells expressing SV40 TAg and COS-7 cells expressing HIV-1 Tat. Based on these observations, the present study was conducted to reproduce an *in vitro* model in COS-7 cells transfected with the JCPyV archetype strain in order to study JCPyV DNA replication and analyze NCCR rearrangements during the viral life cycle. The efficiency of JCPyV replication was evaluated by quantitative PCR (Q-PCR) and by hemagglutination (HA) assay after transfection. In parallel, sequence analysis of JCPyV NCCR was performed. JCPyV efficiently replicated in kidney-derived COS-7 cells, as demonstrated by a progressive increase in viral load and virion particle production after transfection. The archetypal structure of NCCR was maintained during the viral cycle, but two characteristic point mutations were detected 28 days after transfection. This model is a useful tool for analyzing NCCR rearrangements during *in vitro* replication in cells that are sites of viral persistence, such as tubular epithelial cells of the kidney.

## Introduction

John Cunningham virus (JCPyV) is a member of the family *Polyomaviridae*, and it has a small circular double-stranded DNA genome. JCPyV was isolated in 1971 from the brain of a patient with Hodgkin’s disease, and it is the etiological agent of the progressive multifocal leukoencephalopathy (PML), a demyelinating disease of the brain [1].

The JCPyV genome contains two protein-coding regions (early and late), which are transcribed in opposite directions starting from a common non-coding control region (NCCR), the most variable portion of the viral genome. The NCCR contains promoter/enhancer elements and the origin of viral DNA replication [2].

The early region encodes the regulatory proteins large T antigen (TAg) and small t antigen (tAg). During early phase, TAg accumulates and directs initiation of viral DNA replication and the transcriptional switch from early to late gene expression at the onset of the late phase [3]. The late region encodes the viral capsid proteins VP1, VP2, and VP3 and a small regulatory protein, agnoprotein, which is an important factor in the regulation of the viral life cycle. Finally, a gene encoding microRNAs has been identified in the distal part of TAg gene [4].

The archetype NCCR (CY strain) is divided into six regions, named box A (36 bp), box B (23 bp), box C (55 bp), box D (66 bp), box E (18 bp), and box F (69 bp) [5]. Each region contains binding sites for host transcription factors involved in viral early and late transcription. These binding sites undergo deletion and enhancement processes that generate variants that can upregulate viral expression at specific anatomical sites [6, 7]. JCPyV with the archetype sequence, which is found in the kidney and urine, is not associated with PML and is not infectious in tissue culture models. Prototype NCCRs are variants isolated from tissues of patients with PML, and they are named based on the hypothesis that the prototypes result from a rearrangement of the archetype sequence [8]. The original prototype is the Mad-1 isolate, which contains a 98-bp tandem repeat (A-C-E-A-C-E-F) [2, 9].

PML was originally recognized as a rare complication of hematological malignancies or systemic inflammatory disorders; however, a dramatic 50-fold increase in the incidence occurred with the human immunodeficiency virus (HIV) epidemic. Moreover, PML can be seen after organ and stem cell transplantation and, recently, in patients under treatment with immunomodulatory compounds such as monoclonal antibodies (mAbs) [10–12].

Infection with JCPyV is widespread in the general population, with more than 80% being seropositive by adulthood [13]. Nevertheless, the mode of transmission is not yet well defined, although the presence of JCPyV DNA in B cells and stromal cells of the tonsils and oropharynx supports the proposal that tonsils may serve as an initial site of viral infection [14]. Virus might enter the mouth or nose through close interpersonal contact or via fomites, and it presumably spreads by the hematogenous route from the primary site of infection to secondary sites such as kidneys, lymphoid tissues, and brain to establish focal areas of infection or persistence [15–17].

JCPyV is a neurotropic virus; nevertheless, it is still incompletely understood how the virus infects the central nervous system (CNS). There are two possibilities: JCPyV infects the CNS in cases where the immune response is altered; alternatively, the virus infects the CNS and persists there for many years. When alteration of the immune system occurs, viral infection emerges [12].

Although rearrangement of the NCCR of archetype JCPyV is thought to be an important event in the pathogenesis of PML, little is known regarding what induces this rearrangement [18]. It is not yet understood where these rearrangements take place, if they occur in latent cells (bone marrow, precursors of hematopoietic CD34^+^ cells, B cells) or in the brain, during primary or persistent infection. Although various amounts of JCPyV DNA can be found in different blood cell types, there is no direct evidence of productive infection or the presence of viral RNA, proteins or virions [19].

The host cell range of archetype JCPyV is very restricted in cultured cells. Although human primary oligodendrocytes would be the most pathophysiologically relevant *in vitro* model for PML, these cells are difficult to obtain and propagate. Previous reports have demonstrated that archetype JCPyV replicates efficiently in simian-kidney-derived COS-7 cells expressing simian virus 40 (SV40) T antigen and in COS-7 cells expressing HIV-1 Tat [20, 21]. It is also important to know that other JCPyV strains, such as Mad-4, replicate efficiently in simian-kidney-derived COS-7 cells [22], and other cell lines, such as the human embryonic kidney (HEK) cell line 293TT [23] and the human fetal glial cell line SVG, have been employed to study the virus [24]. Although clearly different from primary oligodendrocytes, these cell lines support rapid replication of JCPyV.

The focus of our research was to validate an *in vitro* model in simian-kidney-derived COS-7 cells to study the growth characteristics of JCPyV and to analyze possible NCCR rearrangements that occur after transfection.

## Materials and methods

### Cell line

COS-7 cells were obtained from the ATTC (ATCC® CRL-1651™). COS-7 is a derivative of CV-1(ATCC^®^ CCL-70™), a cell line established from the kidney of an African green monkey that was transformed with an origin-defective mutant of SV40 [25]. Dulbecco’s modified Eagle medium (DMEM) supplemented with 100 U of penicillin, 100 μl of streptomycin per ml, (Sigma-Aldrich S.r.l., Milano, Italia) and fetal bovine serum (FBS) (10%) was used as maintenance medium for the cell line. The cells were incubated at 37 °C in the presence of 5% CO_2_ and propagated at a ratio of 1:4 or 1:8.

### Transformation of competent bacteria

The pCY/cl1 plasmid, which carries the archetype JC genome sequence, was purchased from ATCC (ATCC® VRMC-1™). The JCPyV DNA was recovered for BamHI-digested pCY/cl1 plasmid and the JC linear DNA was gel-extracted and purified using a GenepHlow ™ DNA Cleanup Maxi Kit (Geneaid Biotech Ltd., New Taipei City, Taiwan). The DNA was quantified, and 1 μg was used for transfection experiments.

### Transfection

COS-7 cells were plated with a density of 7.5 ×10^4^ and grown for 24 h in complete growth medium in order to reach 50-70% confluence on the day of transfection. The cells were then transfected with 1 µg of JCPyV CY strain DNA following the specifications of the Xfect ™ Transfection Reagent kit (Clontech Laboratories, Inc., Mountain View, CA, USA). The cells were incubated at 37 °C for 4 hours with the transfection mixture. After two washes with phosphate-buffered saline (PBS), the cells were incubated with complete culture medium for the time course experiment. After two days of incubation, the cells were transferred to a 34-ml flask and then continuously cultured in the maintenance medium with transfer at a split ratio of 1:3/1:4 every 3 or 4 days. Supernatant and cellular fractions were harvested and stored two times a week until 35 days post-transfection (p.t.).

### Extraction of viral DNA from COS-7 cells and from supernatants

The total DNA was extracted from 1 × 10^6^ COS-7 cells using a QIAmp® DNA Mini Kit (QIAGEN S.p.A., Milan, Italy), following the instructions provided by the manufacturer. Once extracted, the DNA was stored at -20 °C until use.

The culture medium from COS-7 cells was initially subjected to six cycles of freezing and thawing and then centrifuged at 2000 rpm for 10 minutes, and the resulting supernatant was used directly in molecular biology assays.

### Hemagglutination (HA) assay

As JCPyV capsids have the property of agglutinating human type O erythrocytes and the HA assay has traditionally been used to determine virus titer [21], the cells were subjected to freeze-thaw cycles. The cell lysates were treated with 50 μg of neuraminidase (Type V; Sigma-Aldrich) per ml at 37 °C overnight, incubated at 56 °C for 30 min, and centrifuged at 200 × *g* for 10 min at 4 °C. The supernatant was serially diluted with 50 μl of PBS in a 96-well round microplate (Costar, Cambridge, MA, USA). The sample was mixed with 50 μl of 0.5% human type O erythrocytes and was further incubated for 3 h at 4 °C. The HA titer was defined as the reciprocal of the highest dilution of virus suspension at which complete HA was observed. The HA activity at the endpoint dilution was considered 1 hemagglutination unit (HAU).

### Quantitative PCR (Q-PCR)

Extracted DNA was analyzed using Q-PCR for the detection and quantification of the JCPyV genome using a 7300 Real-Time PCR System (Applied Biosystems, USA), following a published protocol [26]. Each sample was analyzed in triplicate, and the viral loads were given as the mean of at least three positive reactions. Standard precautions designed to prevent contamination were followed, and a negative control was included in each run. Viral DNA was quantified using a standard curve consisting of serial dilutions of a plasmid containing the entire JCPyV genome with a known titer (range, 10^5^ gEq/ml–10^2^ gEq/ml). The amount of cellular DNA was quantified simultaneously using a SYBR GREEN PCR for the housekeeping β-globin gene [27] and used to normalize the JCPyV DNA. The data were expressed as genome equivalents (gEq) of viral DNA per cell based on DNA content (gEq/cell) for the COS-7 cells and as genome equivalents (gEq) of viral DNA per milliliter (gEq/ml) for the supernatants.

### PCR for JCPyV NCCR

The JCpyV NCCR genomic sequence was amplified using two primers that flank two invariant regions [6]. The primers generated a fragment of 308 bp [28, 29]. Standard precautions were adopted in order to prevent contamination, and a negative control was included in each set of experiments [30]. PCR products were detected by electrophoresis on an ethidium-bromide-stained 2% agarose gel and visualized under UV light.

### Sequencing of the JCPyV NCCR

PCR products corresponding to the JCPyV NCCR region were purified prior to sequencing [6]. DNA sequencing was performed by a professional service (BioFab research s.r.l., Rome, Italy). All sequences obtained from NCCR amplicons were compared to the NCCR of the JCPyV prototype Mad-1 (GenBank: J02227) and to that of the archetype CY (GenBank: AB038249.1). Sequence alignments were performed with Clustal W2 at the EMBL-EBI website using default parameters (ClustalW2-multiple sequence alignment 1) [31].

### Data analysis

Data were expressed as the median and range or as the mean ± standard deviation, as appropriate. Data were analyzed by Student’s *t*-test. *P* < 0.05 was considered statistically significant.

## Results

### Replication efficiency of JCPyV CY in COS-7 cells

In order to evaluate the efficiency of JCPyV replication, COS-7 cells were transfected with 1 µg of DNA of the JCPyV CY archetype strain using an Xfect™ Transfection Reagent kit (Clontech Laboratories, Inc., Mountain View, CA, USA). The transfected cells were grown in maintenance medium for the time course experiment and serially transferred at a split ratio of 1:3 or 1:4 every 3 or 4 days. On day 14, a rounding of the cells started to appear in the cells transfected with the JCPyV CY archetype strain.

Two times a week until day 35 p.t., cells and supernatants were harvested. Samples taken 2 days (T2) after transfection (T0) represented input DNA. In total, the samples were collected at 4 (T4), 7 (T7), 11 (T11), 14 (T14), 18 (T18), 21 (T21), 25 (T25), 28 (T28) and 35 (T35) days p.t.

Intracellular viral DNA was extracted from 1 × 10^6^ COS-7 cells after transfection at the selected sampling time (T2-T35) and used in a quantitative PCR (Q-PCR) assay for JCV TAg in order to evaluate the efficiency of JCPyV replication.

**Fig. 1:**
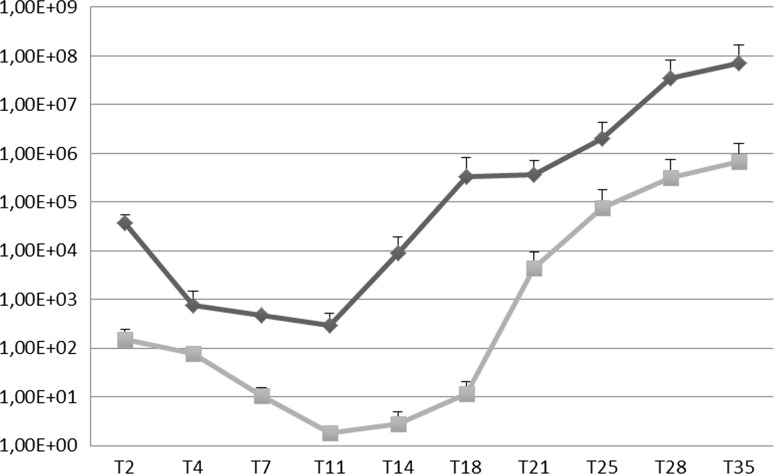
Replication efficiency of JCPyV CY in COS-7 cells and in supernatants. The efficiency of JCPyV replication was assessed at selected sampling times after transfection from 48 hours (T2) p.t. until 35 days (T35). JCPyV DNA was quantified by Q-PCR. The increase in JCPyV replication in COS-7 cells (squares) and in the supernatant (diamonds) during the transfection experiments is shown. Data are expressed as the mean of three independent experiments, and error bars indicate standard deviations. gEq/cell, genome equivalents of viral DNA per cell in COS-7 cells; gEq/ml, genome equivalents of viral DNA per milliliter in the supernatants. T2, 2 days p.t.; T4, 4 days p.t.; T7, 7 days p.t.; T11, 11 days p.t.; T14, 14 days p.t; T18. 18 days p.t.; T21, 21 days p.t.; T25, 25 days p.t.; T28, 28 days p.t.; T35, 35 days p.t

Results of Q-PCR obtained from three independent experiments revealed that JCPyV efficiently replicated in COS-7 cells. In fact, the average JCPyV viral load at T2 was 1.50 × 10^2^ gEq/cell. A decrease in viral replication was observed during the first 18 days p.t. with a progressive increase at T21 (4.49 × 10^3^ gEq/cell), T25 (7.82 × 10^4^ gEq/cell), T28 (3.19 × 10^5^ gEq/cell) and T35, when the JCPyV viral load reached the maximum value of 6.66 × 10^5^ gEq/cell (Fig. 1).

In parallel, JCPyV replication was also evaluated by measuring viral DNA in the supernatants, which were harvested at the same cell sampling times (T2-T35) and subjected to six cycles of freezing and thawing before being assayed by Q-PCR. At two days p.t. (T2), the culture medium showed a JCPyV load average of 3.68 × 10^4^ gEq/ml. The trend of the JCPyV load in the supernatant was essentially the same as that observed in the cells, with a decrease of viral replication in the first 14 days p.t. (Fig. 1). The amount of viral DNA progressively increased for 18 days (T18) (3.38 × 10^5^ gEq/ml), reaching a maximum at 35 days p.t. (T35), with an average value of 7.07 × 10^7^ gEq/ml (Fig. 1).

Comparing the intracellular JCPyV DNA loads (gEq/cell) with those found in the supernatants (gEq/ml), it was observed that JCPyV replication showed the same trend during the sampling period (T2-T35). The efficiency of JCPyV replication during the time course experiment was also estimated by hemagglutination (HA) assay, which is traditionally employed for quantitation of JCPyV. The JCPyV major capsid protein, VP-1, is responsible for red blood cell (RBC) agglutination. The supernatants that were utilized for Q-PCR were assayed for hemagglutination (HA), with the results expressed as a titer. The data showed that the HA titer changed with time. In the archetype-transfected cultures, HA began to be detected on day 14 (T14) (HA = 2) and reached the highest titer on day 35 (T35) (HA = 128).

**Fig. 2:**
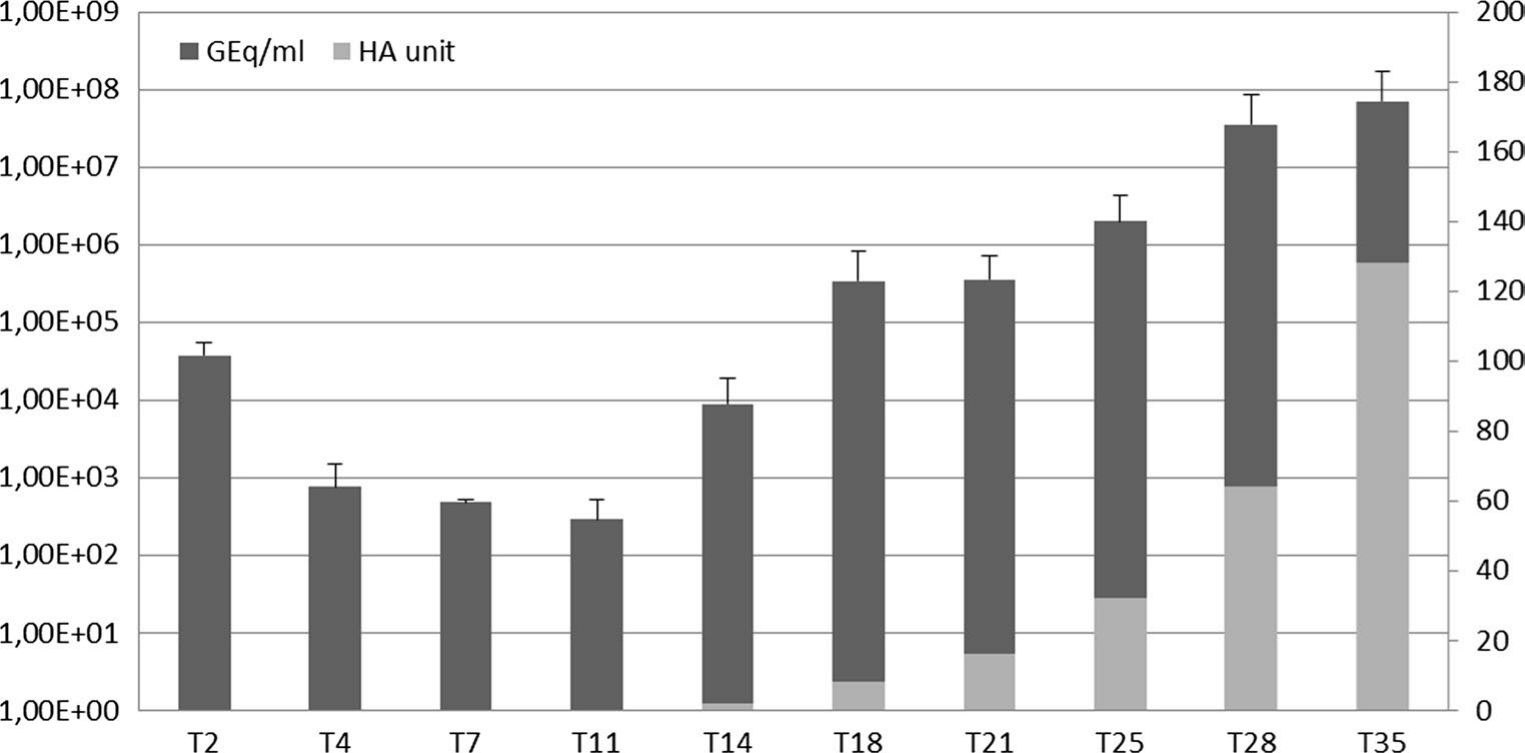
Analysis of HA and Q-PCR data employed for JCPyV quantification. The data represent two independent experiments comparing Q-PCR and the HA assay for the *in vitro* quantification of JCPyV. Samples were tested in triplicate for each run of Q-PCR. Error bars indicate standard deviations. gEq/ml, genome equivalents of viral DNA per milliliter in the supernatants; HA, HA titer; T2, 2 days p.t.; T4, 4 days p.t.; T7, 7 days p.t.; T11, 11 days p.t.; T14, 14 days p.t; T18. 18 days p.t.; T21, 21 days p.t.; T25, 25 days p.t.; T28, 28 days p.t.; T35, 35 days p.t

The HA titer and Q-PCR values progressively increased during the course of transfection, although a quantitative discrepancy between the HA titer and the Q-PCR data was found: the HA titers were low until 14 days p.t. (T14) despite the presence of an average viral load detected by the real-time assays. From T18 to T35, the HA titer and Q-PCR values progressively increased (Fig. 2).

### Sequence analysis of JCPyV NCCR in cells and supernatants at various sampling times

In order to evaluate whether the JCPyV CY archetype strain undergoes rearrangements after being introduced into the permissive cell line COS-7, sequence analysis of the NCCR was performed.

Viral DNA extracted from COS-7 cells and supernatants, harvested at various sampling times post-transfection (T2-T35), was used in a PCR employing two pairs of primers that anneal to the invariant regions flanking JCV NCCR. PCR products were purified and sequenced as described in Materials and methods.

**Fig. 3:**
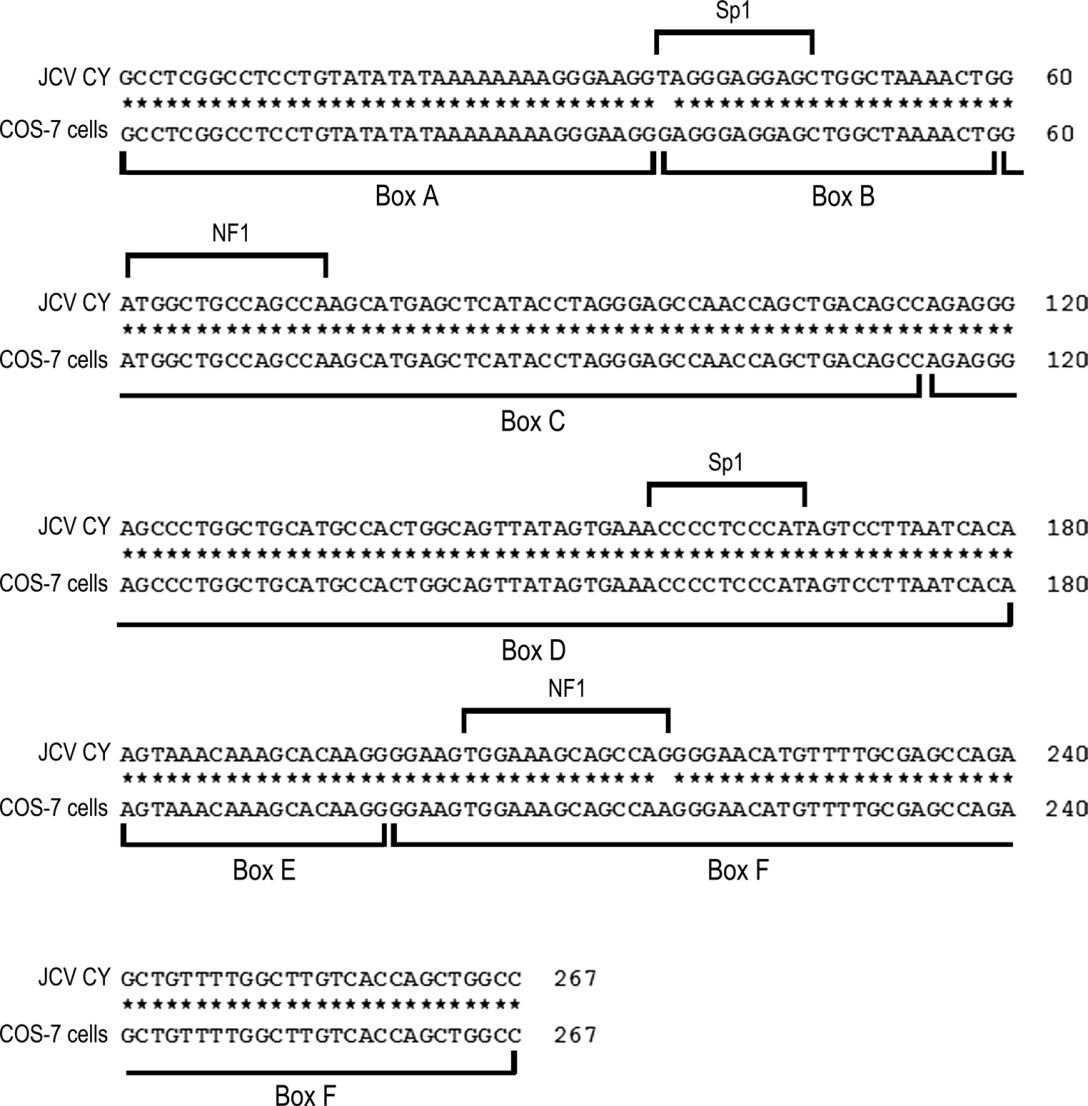
Sequences of the NCCR of JCPyV found in samples of cells and supernatants from renal cell line COS-7 at various sampling times post-tranfection. An alignment is shown between the nucleotide sequence of the archetype NCCR (CY strain), isolated by Yogo and colleagues in 1990 [5], and that obtained from the sequencing of viral DNA extracted from COS-7 cells and from supernatants after transfection. The NCCR of the JCPyV CY strain is generally divided into six regions, named box (A [25 bp], B [23 bp], C [55 bp], D [66 bp], E [18 bp] and F [69 bp]), which are shown below the sequence alignment. The 37 T-to-G nucleotide transversion within the binding site for the cellular transcription factor Spi-B in box B and the 217 G-to-A nucleotide transition in box F at the level of the binding site for the cellular transcription factor NF-1 are shown

Within cell and supernatant samples, the sequence analysis of JCV NCCR showed an archetype-CY-like structural organization during viral growth in COS-7 cells until 25 days post-transfection (T25), with the presence of box A (36 bp), box B (23 bp), box C (55 bp), box D (66 bp), box E (18 bp) and box F (69 bp) (Fig. 3).

Consequently, binding sites for some cell factors involved in viral transcription, such as Tst-1 in box A, Sp1 in box B and NF-1, CRE, and up-TAR in box C, were maintained (Fig. 3). Finally, five or six point mutations were found in the different sequenced samples, but they did not involve known binding sites for transcription factors (data not shown).

The analysis of cell and supernatant samples from T28 and T35 revealed an archetype-CY-like NCCR with two characteristic point mutations: the 37T-to-G nucleotide transversion within the binding site for the cellular transcription factor Spi-B in box B and the 217G-to-A nucleotide transition in box F at the level of the binding site for the cellular transcription factor NF-1 (Fig. 3).

## Discussion

The natural course of JCPyV infection is life-long persistence in the case of immunocompetent hosts, but the mechanism by which JCPyV establishes this delicate equilibrium within the host remains poorly defined. JCPyV infection is very common and sero-epidemiological studies have shown that initial infection occurs early in life [13]. In fact, archetype JCPyV (CY strain) infects children and establishes asymptomatic and persistent infection in the kidneys, with occasional shedding in urine when low-level replication of persistent virus occurs in tubular epithelial cells [19]. Specific T-cell immunity plays a significant role in controlling JCPyV replication and protection from PML [32]. Archetype JCPyV has also been detected repeatedly in human peripheral blood mononuclear cells (PBMCs) – more specifically, B cells – and it is possible that the virus establishes a subclinical infection in other immune cells, such as bone marrow progenitor cells [33, 34]. The rearranged form of JCPyV (prototype NCCR, also known as PML-type JCV) can be isolated from the brain and lymphocytes in people with and without PML and is typified by the Mad-1 strain of JCPyV [8, 17, 24]. The prototype variant has high replication capacity and has been associated with insufficient or dysregulated T-cell responses [32].

To date, cell culture systems to propagate JCPyV have been very limited in their availability and robustness. Based on this observation, our aim was to produce a model to study the growth characteristics of JCPyV and to analyze the possible NCCR rearrangements that occurred after transfection. The large amount of JCPyV DNA detected during the time course of transfection confirms the ability of JCPyV to replicate in simian-kidney-derived COS-7 cells expressing SV40 TAg. The levels of JCPyV replication showed the same trend in the cells and supernatants, but the amount of viral DNA found in the supernatants was higher than in the cellular fraction. This is due to an efficient release of free virions in the culture medium. Moreover, the detachment of infected cells from the surface of the flasks due to the intensive utilization of cellular resources by JCPyV for its life cycle contributes to the massive presence of viral DNA in the supernatant. Although, it had been demonstrated prior to this study that archetype JCPyV DNA replicates in COS-7 simian kidney cells expressing SV40 TAg and COS-7 cells expressing HIV-1 Tat [20, 21], the novelty of our work is that we monitored JCPyV replication more precisely by Q-PCR during transfection. As described in the results, the HA titer, which is traditionally used to determine virus titer, and Q-PCR values progressively showed the same trend of increase during the course of transfection, although a quantitative discrepancy between the HA titer and Q-PCR data was found, with HA titers remaining low until 14 days p.t. (T14) despite the presence of an average viral load detected by Q-PCR. From T18 to T35, HA titer and Q-PCR values progressively increased. Q-PCR is a more sensitive and reliable method for *in vitro* quantification. As described by Chapagain and colleagues [35], Q-PCR has proven to be an important method for monitoring JCPyV viral load in clinical specimens, and it is a reliable marker for PML prognosis.

The second novel aspect of our work is that we studied the molecular characteristics of the JCPyV NCCR after transfection in an effort to determine whether the archetype strain undergoes rearrangements in the permissive COS-7 cells. The mechanisms that induce rearrangements in the NCCR are still poorly understood. Our results showed that an archetype CY-like structural organization of the NCCR was consistently maintained, with the presence of all binding sites for cellular factors involved in viral transcription in all samples (cells and supernatants) collected from the input JCPyV DNA until 25 days p.t. It should be noted that the archetypal structure of the NCCR was maintained during viral growth, although the archetype generates various PML-type regulatory regions by deletion and amplification during persistence of JCV in patients [5, 20, 36–40]. This could be due to the fact that the transfected cells were grown for a few weeks, a period much shorter than that of the persistence of the CY strain in humans [20]. Surprisingly starting from 28 days post-transfection, analysis of the NCCR revealed an archetype-CY-like structural organization with two characteristic point mutations: the 37T-to-G nucleotide transversion within the binding site for the cellular transcription factor Spi-B in box B and the 217G-to-A nucleotide transition in box F at the level of the binding site for the cellular transcription factor NF-1. For the first time, these point mutations were detected in an NCCR structure derived from an *in vitro* replication. They were previously reported in urine of immunocompetent individuals [41] and in urine and PBMCs of multiple sclerosis patients undergoing natalizumab treatment [42]. It should be noted that the 37T-to-G nucleotide transversion in box B is located within the binding site for the cellular transcription factor Spi-B, which can play a crucial role in viral replication and neurovirulence. It is known that Spi-B carries out two functions in JCPyV replication. The first one is the activation of the JCPyV early promoter in both glial and non-glial cells in relation to its binding to box B site, and the second one is the TAg-mediated transactivation of viral early genes in relation to its binding to the site located in box D [7, 43]. Regarding the 217G-to-A nucleotide transition in box F within the binding site for the cellular transcription factor NF-1, it has been reported that NF-1 increases the expression of early and late JCPyV genes in glial cells, which are permissive to viral replication [42, 44, 45].

In conclusion, the present study confirms that this *in vitro* system can be used to grow the CY archetype form of JCPyV. This model could be useful for investigating the natural history of infection and developing means to prevent transmission and reactivation. It can also be used to study the possibility of JCPyV NCCR rearrangement at sites of viral persistence, such us the tubular epithelia of kidneys and to understand the effect of NCCR rearrangements on early and late gene expression.

It is important to understand the nature of JCPyV infection in cell types supporting productive viral infection and to determine which cell types are involved in the process of JCPyV rearrangement. This is the missing piece in the pathogenesis puzzle of PML: in fact, the importance of NCCR rearrangements in the onset of PML has been demonstrated by the discovery of highly rearranged sequences in patients with this condition. However, it is not yet understood where and when these rearrangements occur, or if they occur during primary infection or during the period of persistence of the virus in the host.
